# A Primer and Literature Review on Internal and External Retention Mechanisms for Catheter Fixation

**DOI:** 10.7759/cureus.24616

**Published:** 2022-04-30

**Authors:** Christopher M Stevens, Kevin Malone, Deven Champaneri, Nick Gavin, Daniel Harper

**Affiliations:** 1 Interventional Radiology, Louisiana State University Health Sciences Center, Shreveport, USA; 2 Biomedical Engineering, Louisiana State University Health Sciences Center, Shreveport, USA; 3 Radiology, Medical University of South Carolina, Charleston, USA; 4 Radiology, Virginia College of Osteopathic Medicine (VCOM) - Carolinas, Spartanburg, USA

**Keywords:** pigtail catheter, malecot catheter, foley catheter, retention mechanisms, catheter migration

## Abstract

Although catheters are commonplace in hospital settings, there is scarce literature discussing the internal and external retention mechanisms used to aid in catheter fixation. Additionally, exact definitions and detailed information on internal and external retention mechanisms are almost non-existent in the literature.

This article serves three primary purposes. The first purpose is to define internal and external catheter retention mechanisms, describe how they work, and provide examples of each that are routinely used in healthcare settings. The second goal of this paper is to provide a literature review comparing various aspects of the different types of internal and external catheter retention mechanisms discussed in the paper, including performance variance and the advantages and disadvantages of each. The third aim of this article is to provide a brief overview of catheter dislodgment, including the rates at which this occurs, the problems that can arise, and the best treatment option when this does occur.

## Introduction and background

The word “catheter” is derived from the Greek word καθίημι (kathíēmi), meaning “to let down or descend” [1]. By definition, a catheter is a segment of tubing inserted into the body - through natural orifices or percutaneously - creating an opening to administer a drug, distend a passageway, or remove fluid. One of the earliest records of catheter use dates back to around 1500 BCE when Egyptians used drainage catheters made from straw, reeds, curled-up palm leaves, and bronze tubes to relieve urinary retention in males [1]. Today, catheters are utilized in nearly every hospital setting.

An essential, yet under-looked, aspect of catheter use is the secure fixation of the catheter. Fixation of catheters is done to prevent catheter dislodgment, a complication that can cause pain, discomfort, local tissue damage, and the spilling of infected contents into the surrounding areas [2]. To enhance catheter fixation, internal and external retention mechanisms are commonly used, but literature describing their concept and usage is scarce. 

This article has three primary purposes. The first is to define and elaborate on the various aspects of internal and external catheter retention mechanisms, including the definition for each, how they work, how they are different from one another, and provide examples of each that are routinely used in healthcare settings. The second is to provide literature reviews for both internal and external catheter retention mechanisms that evaluate the performance variance, advantages, and disadvantages of each. The third aim of this article is to provide a brief overview of catheter dislodgment, including the rates at which this occurs, problems that can arise, and the best treatment option for when this does happen.

## Review

Difference between internal and external retention mechanisms

While both internal and external retention mechanisms aid in the stability of drainage catheters by helping prevent dislodgment, they are two distinct entities that work independently of each other. The two main characteristics distinguishing internal and external retention mechanisms are location and configuration. Location is in reference to the patient while configuration is in relation to the catheter itself. 

For location, the terms “internal” and “external” can be thought of as being in relation to inside or outside the body of the patient, meaning that internal retention mechanisms, such as pigtail cope loops, exist within the body, while external retention mechanisms, such as sutures, exist on the outer surface of the body. The distal end of a catheter is the end inserted furthest into the body cavity when placing a catheter, making it the part of the catheter that houses the internal retention mechanism. Contrary to this, the proximal end of the catheter is the location where an external retention mechanism is located. For configuration, internal retention mechanisms are directly built into the catheter and do not exist as an independent entity of it. However, external retention mechanisms are a separate entity and have to be applied to the catheter by the user since they are not part of the catheter itself.

Internal retention mechanisms (mushroom tips, balloons, and pigtails)

Catheters may have an internal retention mechanism to aid in catheter stability, particularly when placed in moving organs or spaces, e.g., kidneys, gallbladders, or stomachs. Commonly used internal retention mechanisms include mushroom tips (Pezzer and Malecot catheters), balloons (Foley catheter), and pigtails with a suture locking mechanism (Cope loop).

**Figure 1:**
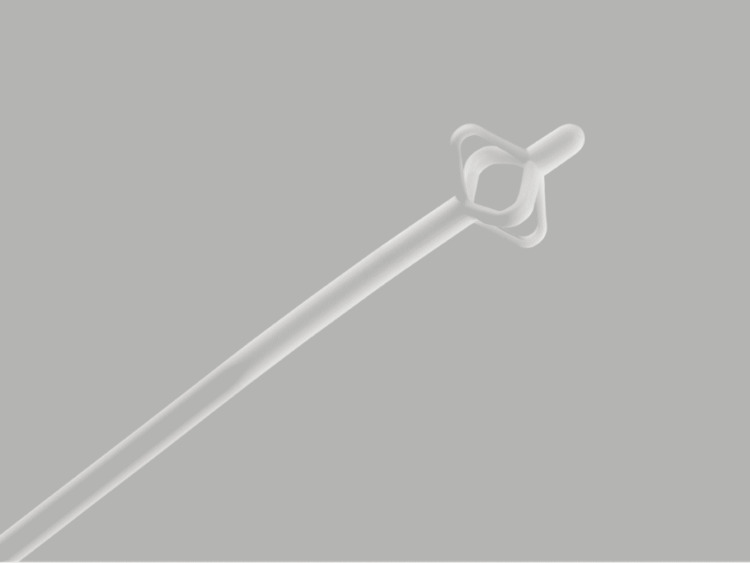
Image of the distal end of a Malecot catheter Image of the distal end of Malecot catheter with expanded wings creating the appearance of a mushroom Image credit: Cook Medical, Silicone Malecot Catheter, https://www.cookmedical.com/products/uro_0838_webds/

Malecot catheters are used for various types of drainages and are the preferred choice for percutaneous nephrostomy [3-4]. The internal retention mechanism for Malecot catheters is defined by the shape of the catheter’s distal end. The distal end (tip) is shaped similar to a mushroom due to the presence of expandable, circular wings bilaterally, which increase the stability of the catheter (Figure 1) [5-6].

The Foley catheter is perhaps the most prevalent type of catheter used in healthcare. The most common use for Foley catheters is bladder drainage via insertion through the urethra [1]. While Foley catheters are used worldwide at a high rate, their usage is generally short and strict due to complications resulting from their long-term use [7]. As a general rule, Foley catheters are only to be left in the patient for a maximum of 30 days, as the patient’s chances of acquiring an infection increase substantially after this time [7-8].

**Figure 2:**
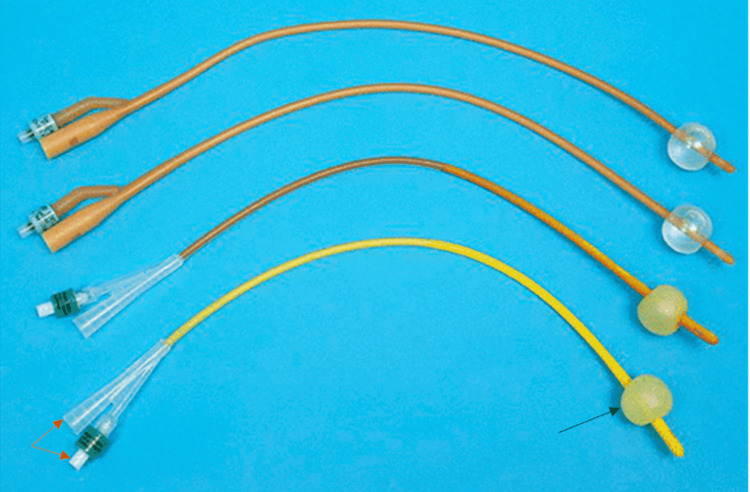
Image of Foley Catheters Image of four Foley Catheters, each with two channels (red arrows on the bottom catheter) at the proximal end and an inflated balloon (black arrow on the bottom catheter) at the distal end to aid in catheter stabilization Image credit: Continence Product Advisor, Indwelling Catheters, continenceproductadvisor.org

A balloon, located at the tip of the catheter and just proximal to the drainage eyelets, serves as the internal retention mechanism for Foley catheters (Figure 2) [8-9]. A Foley catheter usually has two channels at the proximal end (which remains outside the body), a dominant drainage channel that removes urine and a lesser inflation channel where sterile water from a syringe is administered for inflation of the balloon [9]. Once the catheter is inserted through the urethra and placed in the patient’s bladder, the balloon is inflated (Figure 2). The inflated balloon aids in stabilizing the catheter inside the bladder, thereby allowing the catheter to remain in place to facilitate proper drainage of urine.

**Figure 3:**
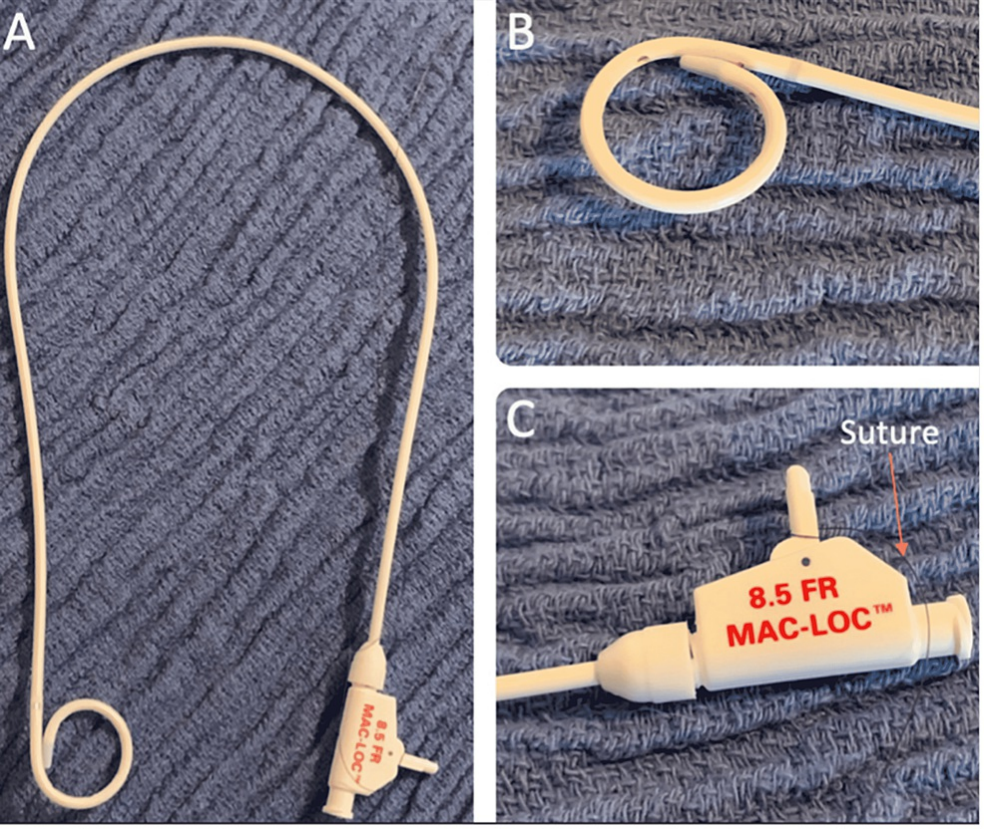
Images of a Pigtail Cope Loop Catheter A. Whole pigtail Cope loop catheter B. Distal end of the pigtail Cope loop catheter (resides in a pigtail loop that aids in fixation of the catheter) C. Proximal end of the pigtail Cope loop catheter (contains the suture that is pulled to create the pigtail loop at the distal end of the catheter)

Pigtail Cope loop catheters are routinely used to evacuate pleural fluid collections [10]. The pigtail Cope loop catheter’s inner retention mechanism involves a full-length suture that internally connects the catheter’s distal end to the catheter’s proximal end at the locking hub. Once the catheter is inserted into the body and in the correct position, the suture is pulled at the proximal end, creating a pigtail loop in the distal end (Figure 3). This pigtail loop aids in stabilizing the pigtail catheter by not allowing the catheter to be easily pulled out through the entry tract.

External retention mechanisms (sutures and sutureless adhesive devices)

**Figure 4:**
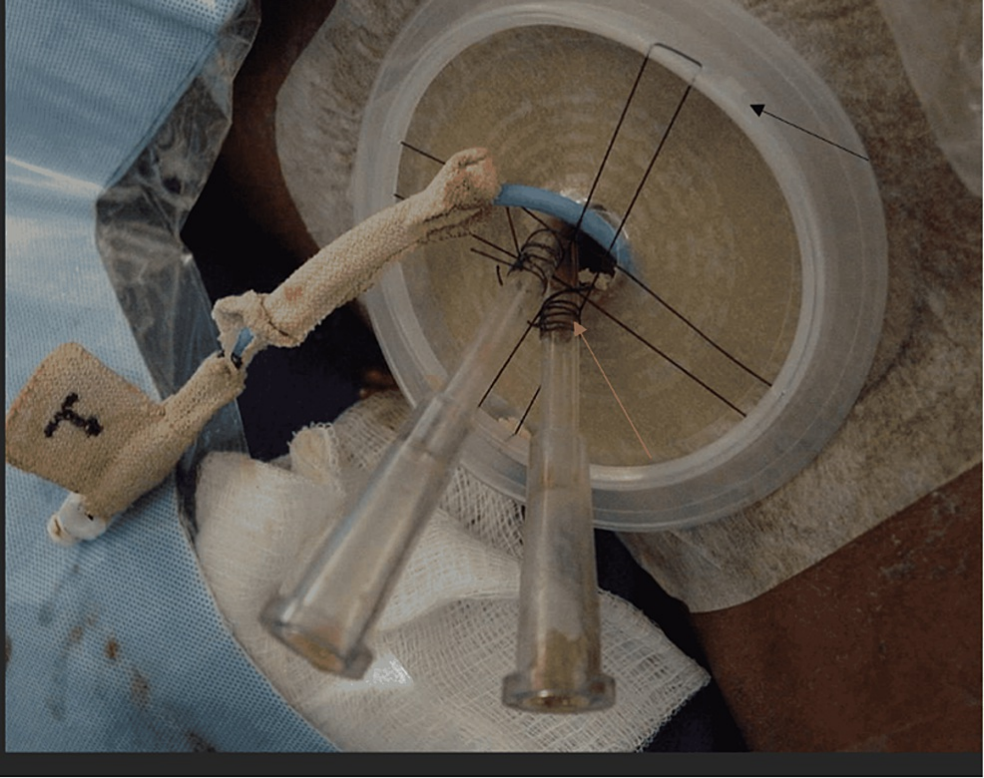
Image Showing Sutures Being Used for Catheter Securement Image showing sutures (orange arrow) used for external catheter securement to skin. In the figure, the sutures are connected to an ostomy barrier (black arrow) rather than puncturing the skin to decrease the risk of infection. Image obtained with permission from Dr. Horacio D'Agostino.

**Figure 5:**
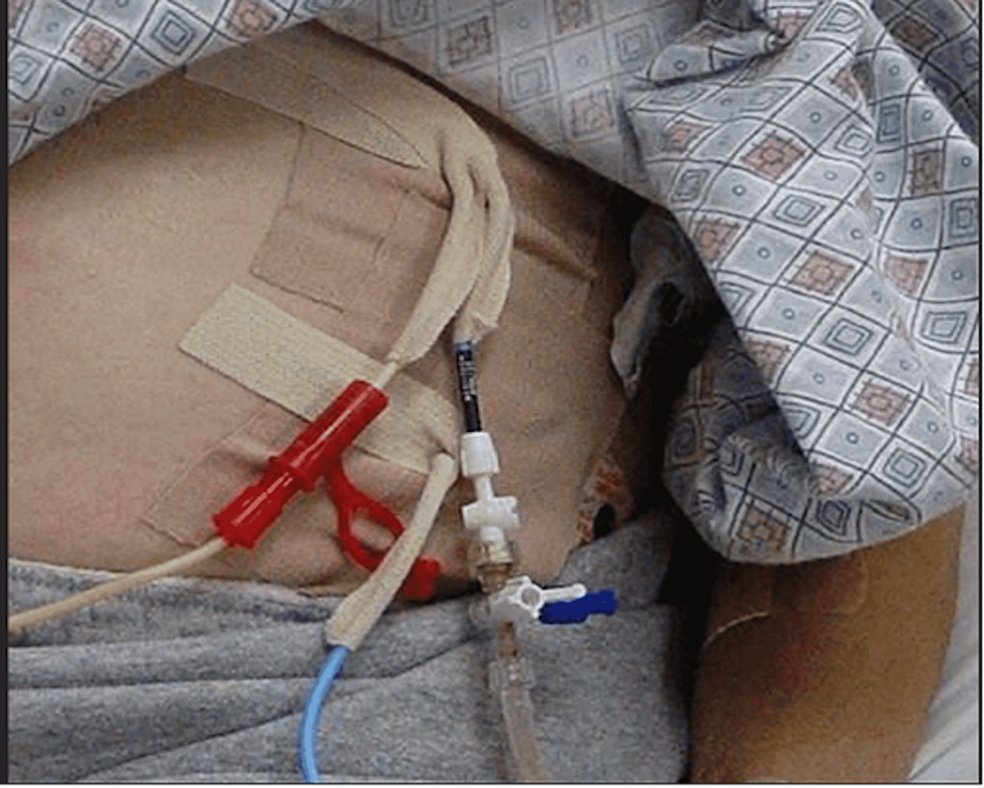
Image Showing Tape Being Used for External Catheter Securement to Skin Image obtained with permission from Dr. Horacio D'Agostino.

**Figure 6:**
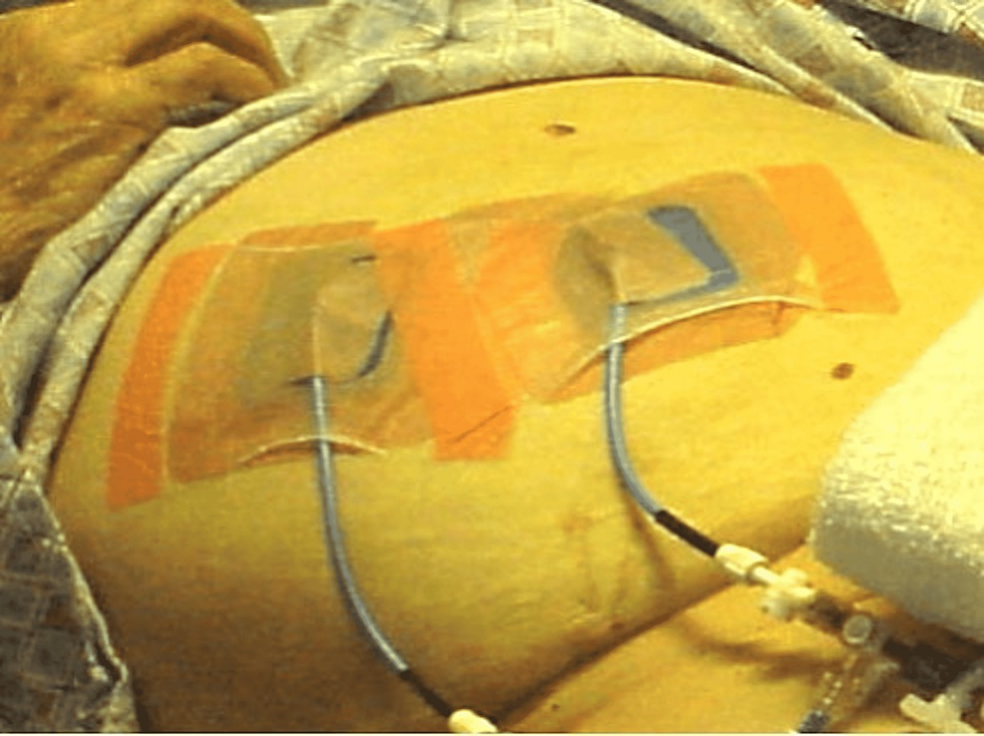
Image Showing Two Adhesive Films Used for External Catheter Securement to Skin Image obtained with permission from Dr. Horacio D'Agostino.

External retention mechanisms for catheters consist of sutures and sutureless adhesive devices applied to the skin. Sutures are primarily known for their skin closure use but are also an effective means of securing catheters in a fixed position (Figure 4) [11]. Sutureless devices, such as tape (Figure 5) and adhesive film (Figure 6), secure catheters to the skin and are intended to decrease the risks associated with sutures [12].

According to previous reports and guidelines [12-13], for external retention devices to be rendered effective, they must meet the following seven criteria:

1. Securement for the intended time of wear (an increased dressing-change frequency has been associated with an increase in infection, driving the need for longer wear times)

2. Prevention of catheter migration

3. Maintenance of skin integrity around the insertion site (skin damage requires action up to and including relocation of the catheter)

4. Ease of application and removal

5. Universal use (devices must be usable with more than one size or brand of catheter)

6. Compatibility with standard skin preps and other devices

7. Compatibility with monitoring of the insertion site and the delivery of therapies

Literature review for internal retention mechanisms

**Figure 7:**
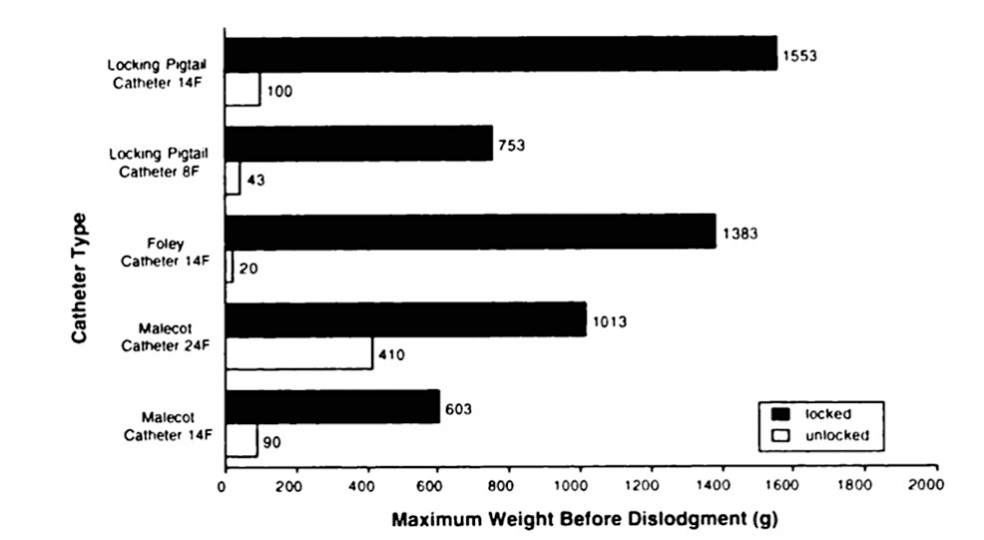
Comparison of the Maximum Weight Supported by Each Type of Catheter (Pigtail, Foley, Malecot) Bar graph indicating that the Foley and pigtail catheters performed the best when tested for resistance to dislodgment [14] Image obtained with permission from Dr. Horacio D'Agostino

Literature describing the usage and performance of the internal retention mechanisms discussed in the present article is exceptionally scarce. In our search, only one paper was found to have adequate information regarding these three different types of internal retention mechanisms. This in vitro study evaluated the resistance to dislodgment of the three different retention mechanisms discussed and found that the balloon and suture locking mechanisms were the most effective (Figure 7) [14].

Literature review for external retention mechanisms

Clinicians have a wide array of external catheter securement devices they can choose from. External securement of catheters became prominent in the 1980s with the initial use of simple tape or gauze tape [15]. Multiple studies have shown that using non-sterile tape alone results in higher catheter-related complication rates than other means of catheter securement [16-18]. Due to these complications, non-sterile tape alone is not considered a primary means of external catheter securement. However, using non-sterile tape alone for external catheter securement is still commonplace, mostly in low-income regions due to the commercially low price of tape, with approximately one in five catheters worldwide externally secured with non-sterile tape alone [19-20]. While non-sterile tape alone seems to not be an efficient method for external catheter fixation, non-sterile tape in addition to other securement methods has shown positive outcomes. Crowell et al. reported fewer rates of catheter occlusion when primary dressings were reinforced with non-sterile tape [21]. On two separate occasions, a decreased rate of catheter dislodgment was reported when non-sterile tape was used in conjunction with primary dressings [22-23]. Compared to polyurethane dressings, the use of gauze and tape resulted in lower incidences of catheter-related bloodstream infections, but this may be due to bias [24]. The use of sterile tape can potentially add extra security at the proximal hub of the catheter, but more research needs to be done to confirm this [19].

In the past, sutures were commonly used as an external retention mechanism to secure catheters; however, today, several limitations exist with the use of sutures for external catheter securement, including needlestick injuries and increased risk of infection due to additional puncture sites through the skin [11-12,15,25-26]. Research has also shown sutureless devices to be superior to sutures for protecting against catheter dislodgment by aiding in catheter stabilization [27]. In 2011, a guideline regarding the prevention of intravascular catheter-related infections advocated for the use of sutureless devices over sutures for catheter securement [28]. A literature review published in 2012 concluded that using sutureless adhesive devices reduced the occurrence of infection compared to using sutures that punctured the skin [26]. Krenik et al. designed an external sutureless securement system for peripherally inserted central catheters and had clinicians at 19 different locations test and rate its performance to their current external securement system, which consisted of either sutures or other sutureless devices, on the following criteria: prevention of catheter migration, catheter migration during removal, adhesion time, patient comfort, skin redness, itching, or irritation, and gentleness to skin. Ninety-seven point three percent (97.3%) of the clinicians rated the performance of the novel device as being the same, better, or much better than that of sutures or other securement devices the clinicians currently used [12], thus concluding that the use of sutures resulted in either worse or equivalent outcomes when compared to that of the sutureless novel device. In 2016, the Infusion Nurses Society stated that engineered catheter stabilization devices were superior to both sutures and tape and that sutures and tape were not an effective means of catheter securement [13]. Fujimoto et al. conducted a retrospective cohort study in 2021 that compared the performance difference between traditional skin sutures and GRIP-LOK devices, a sutureless securement device. They found that the GRIP-LOK device was more effective than traditional skin sutures at preventing catheter-related bloodstream infections, catheter site exit infections, and catheter dislodgment in patients using a non-tunneled hemodialysis catheter [29]. Due to the better performance and decreased risk that is seen with the use of sutureless adhesive devices, the use of sutures for catheter securement has decreased [12].

The StatLock stabilization device, a strap-free device frequently used to secure Foley catheters, appears to be the most commonly used sutureless catheter securement device in healthcare [12]. A clinical trial compared the performance of three different sutureless securement devices, nonsterile tape, StatLock, and Hub-Guard, on their ability to sufficiently extend the average survival time of peripheral intravenous (PIV) catheters to allow the implementation of a 96-hour PIV change-protocol. Results showed that the StatLock device produced the greatest survival rate (52%). When using the nonsterile tape or Hub-Guard as securement methods, the survival rates were 8% and 9%, respectively [18]. Yamamoto et al. also found that the use of StatLock securement devices resulted in significantly fewer peripherally inserted central venous catheter-related bloodstream infections compared to the use of sutures [30]. Another clinical trial concluded that the use of transparent dressing and StatLock resulted in a 40% reduction in catheter dislodgment and a 45% reduction in therapy complications compared to tape and transparent dressing [31]. A randomized multicenter clinical trial involving 127 patients found that the use of StatLock resulted in a 45% reduction rate of symptomatic catheter-related urinary tract infections [32]. Sheppard et al. reported an improvement in clinical outcomes and quality of care using StatLock [33]. A 2017 meta-analysis involving 13 randomized controlled trials spanning 1970 patients concluded that the use of StatLock, compared to the use of tape or sutures, resulted in increased patient comfort and decreased catheter-related complications [34].

Dislodgment of catheters 

It is important to note that although internal and external retention devices are designed to promote catheter fixation, catheter dislodgment has been, and is still, a problem experienced by many patients [35-40]. Catheter dislodgment can be attributed to several factors, including patients who pull on the catheter themselves or accidental dislodgment from routine physical activity such as walking or moving [41]. Dislodgment can range in severity, from small, repeated movements or micromotion to large catheter migration in an outward direction or complete dislodgment in severe scenarios [12]. Males may be at increased risk of catheter migration due to naturally having more muscle and hair [35].

The primary treatment method for dislodged catheters includes inserting a new catheter through the original tract or creating a new tract [2,42]. This alone is another reason why catheter dislodgment can be harmful because it requires further exposing the patient to the risks of an additional procedure. Since dislodged catheters can cause severe adverse effects, including damage to the surrounding tissue and spilling of infected contents into the surrounding areas, immediate treatment upon diagnosis of catheter dislodgment is highly recommended [2]. The time interval between the catheter dislodgment and the insertion of a new catheter seems to influence what route is best used to insert the new catheter. If dislodgment is recognized early enough, roughly within the first days of the initial dislodgment, using the original tract to insert the new catheter has a high success rate, being that the short time interval does not allow for the tract to mature [42]. Since reinsertion through the original tract is less invasive, less time-consuming, and usually more comfortable for the patient, it is recommended to use this treatment method if no complications exist [42-43].

Since catheter dislodgment can have these adverse effects and is still a common occurrence seen in many different catheter types (Table 1) [41,44-46], more research and advancements in catheter fixation need to be conducted to counteract this problem. Decreased catheter migration would result in more efficient catheter use and fewer complications experienced by the patient. Just a 10% reduction in dislodgment of PIV catheters has the potential to prevent >30 million PIV reinsertions and failures yearly nationwide [47-48].

**Table 1:** Dislodgment Rate for Enteric Feeding, Biliary Drainage, Percutaneous Nephrostomy, Intravenous, Peritoneal Dialysis, and Long-Term Indwelling Urinary Catheters

Catheter Type	Catheter Dislodgment Rate
Enteric Feeding Catheters	11.9%
Biliary Drainage Catheters	3.4%
Percutaneous Catheter Nephrostomy	15%
Intravenous Catheters	1.8%-24%
Peritoneal Dialysis Catheters	62.4%
Long Term Indwelling Urinary Catheter	12%

Limitations

Due to the scarcity of literature, a detailed and elaborate literature review on internal catheter retention mechanisms was unable to be conducted, with only one article being cited and discussed in this section.

## Conclusions

Though insertion of catheters into patients is one of the most common procedures in healthcare, there is a scarce amount of literature on catheter retention mechanisms that are used to aid in catheter fixation. This paper defined catheter-related retention mechanisms, the difference between internal and external retention mechanisms, and provided examples of commonly used internal and external retention mechanisms. Two separate literature reviews, one evaluating the internal retention mechanisms and the other evaluating the external retention mechanisms discussed in this article, were also conducted. We also sought to highlight the importance of advancements in catheter fixation by acknowledging that catheter dislodgment still exists as a current problem, even with the use of the available retention mechanisms described.

The goal of the literature reviews was to determine if there was a difference in the performance between the mechanisms described. Only one study was found to evaluate the performance of the internal retention mechanisms, and it was concluded that the Foley and pigtail catheters outperformed the Malecot catheter for resistance to dislodgment. The fact that only one article was able to be cited for this literature review also highlights the gap that exists in the literature discussing this topic. The literature review conducted on the external catheter retention mechanisms concluded that suture use for external catheter securement has decreased due to increased rates of infection and needlestick injuries associated with sutures. The use of non-sterile tape alone was also found to be an inefficient means for external catheter securement, despite 20% of globally inserted catheters only using non-sterile tape as its only source of external fixation. However, tape, in conjunction with other securement devices, was an efficient way to secure catheters externally. Sutureless securement devices, such as StatLock and GRIP-LOK, were found to be the most effective means for external catheter securement, compared to sutures and tape, due to their ability to reduce catheter complications.
